# An Improved Postprocessing Method to Mitigate the Macroscopic Cross-Slice *B*_0_ Field Effect on $R_{2}^{*}$ Measurements in the Mouse Brain at 7T

**DOI:** 10.3390/tomography10070081

**Published:** 2024-07-11

**Authors:** Chu-Yu Lee, Daniel R. Thedens, Olivia Lullmann, Emily J. Steinbach, Michelle R. Tamplin, Michael S. Petronek, Isabella M. Grumbach, Bryan G. Allen, Lyndsay A. Harshman, Vincent A. Magnotta

**Affiliations:** 1 Department of Radiology, University of Iowa, Iowa City, IA 52242, USA; chu-yu-lee@uiowa.edu (C.-Y.L.); dan-thedens@uiowa.edu (D.R.T.); 2 Medical Scientist Training Program, University of Cincinnati College of Medicine, Cincinnati, OH 45267, USA; lullmaoa@mail.uc.edu; 3 Stead Family Department of Pediatrics, Division of Pediatric Nephrology, University of Iowa Carver College of Medicine, Iowa City, IA 52242, USA; emily-steinbach@uiowa.edu (E.J.S.); lyndsay-harshman@uiowa.edu (L.A.H.); 4 Division of Cardiovascular Medicine, Abboud Cardiovascular Research Center, Department of Internal Medicine, University of Iowa Carver College of Medicine, Iowa City, IA 52242, USA; michelle-tamplin@uiowa.edu (M.R.T.); isabella-grumbach@uiowa.edu (I.M.G.); 5 Department of Radiation Oncology, Free Radical and Radiation Biology, University of Iowa Carver College of Medicine, Iowa City, IA 52242, USA; michael-petronek@uiowa.edu (M.S.P.); bryan-allen@uiowa.edu (B.G.A.); 6 Iowa City VA Center for the Prevention and Treatment of Visual Loss, Iowa City, IA 52246, USA; 7 Department of Psychiatry, University of Iowa, Iowa City, IA 52242, USA; 8 Department of Biomedical Engineering, University of Iowa, Iowa City, IA 52242, USA

**Keywords:** background gradients, $R_{2}^{*}$, $T_{2}^{*}$, post-processing, noise, gradient-echo, *B*_0_ inhomogeneity, brain, myelin, iron, quantitative MRI

## Abstract

The MR transverse relaxation rate, $R_{2}^{*}$, has been widely used to detect iron and myelin content in tissue. However, it is also sensitive to macroscopic *B*_0_ inhomogeneities. One approach to correct for the *B*_0_ effect is to fit gradient-echo signals with the three-parameter model, a sinc function-weighted monoexponential decay. However, such three-parameter models are subject to increased noise sensitivity. To address this issue, this study presents a two-stage fitting procedure based on the three-parameter model to mitigate the *B*_0_ effect and reduce the noise sensitivity of $R_{2}^{*}$ measurement in the mouse brain at 7T. MRI scans were performed on eight healthy mice. The gradient-echo signals were fitted with the two-stage fitting procedure to generate $R_{2corr\_t}^{*}$. The signals were also fitted with the monoexponential and three-parameter models to generate $R_{2nocorr}^{*}$ and $R_{2corr}^{*}$, respectively. Regions of interest (ROIs), including the corpus callosum, internal capsule, somatosensory cortex, caudo-putamen, thalamus, and lateral ventricle, were selected to evaluate the within-ROI mean and standard deviation (SD) of the $R_{2}^{*}$ measurements. The results showed that the Akaike information criterion of the monoexponential model was significantly reduced by using the three-parameter model in the selected ROIs (*p* = 0.0039–0.0078). However, the within-ROI SD of $R_{2corr}^{*}$ using the three-parameter model was significantly higher than that of the $R_{2nocorr}^{*}$ in the internal capsule, caudo-putamen, and thalamus regions (*p* = 0.0039), a consequence partially due to the increased noise sensitivity of the three-parameter model. With the two-stage fitting procedure, the within-ROI SD of $R_{2corr}^{*}$ was significantly reduced by 7.7–30.2% in all ROIs, except for the somatosensory cortex region with a fast in-plane variation of the *B*_0_ gradient field (*p* = 0.0039–0.0078). These results support the utilization of the two-stage fitting procedure to mitigate the *B*_0_ effect and reduce noise sensitivity for $R_{2}^{*}$ measurement in the mouse brain.

## 1. Introduction

The voxel-wise MR transverse relaxation rate, $R_{2}^{*}$, is sensitive to in vivo iron and myelin levels in the brain [1,2,3]. It has been used broadly to study brain development and neurodegenerative diseases in humans and mouse models associated with alternations in myelin and iron content [4,5,6,7,8,9,10,11]. $R_{2}^{*}$ mapping is typically achieved by acquiring multi-echo gradient echo (GRE) images, followed by voxel-wise mono-exponential fitting of the signal decay. In the presence of iron and myelin, the magnetic susceptibility variations induce microscopic and mesoscopic field inhomogeneities in a voxel, resulting in accelerated signal decay and an increase in $R_{2}^{*}$. However, due to macroscopic voxel sizes, $R_{2}^{*}$ values become inaccurate due to macroscopic field inhomogeneities (Δ*B*_0_), such as regions near air-tissue interfaces. This considerable Δ*B*_0_ effect leads to additional signal decay and overestimation of the $R_{2}^{*}$ [12]. Therefore, correcting for the Δ*B*_0_ effect is necessary for accurate $R_{2}^{*}$ measurement.

The Δ*B*_0_ effect can be mitigated by using a small voxel size on a microscopic or mesoscopic scale, but it is impractical due to the low signal-to-noise ratio (SNR) [12,13]. For GRE images with a high in-plane resolution but thicker slices such as in a typical two-dimensional (2D) acquisition, the Δ*B*_0_ effect is predominant along the z-direction [12,14]. Many methods have been proposed to correct for the z-direction Δ*B*_0_ effect on $R_{2}^{*}$ measurement [14,15,16,17,18,19,20,21,22,23,24,25]. By assuming that the z-direction Δ*B*_0_ is linear, the correction can be performed using one of the following methods: (1) adding a z-direction gradient to compensate for Δ*B*_0_ [14,23]; (2) combining images from multiple acquisitions with incremental z-direction gradients [15,24,25]; or (3) applying a tailored RF excitation pulse [26] to reduce the intra-voxel spin dephasing due to Δ*B*_0_. Alternatively, the correction can be performed through postprocessing [16,17,18,19,20,21,22], which does not require pulse sequence modifications and can be generally applied to multi-echo GRE images. For an ideal slice profile and a linear Δ*B*_0_, the Δ*B*_0_ effect on the measured $R_{2}^{*}$ can be corrected by applying a sinc weighting function to the monoexponential model [17]. The corrected $R_{2}^{*}$ ($R_{2corr}^{*}$) can be obtained by fitting the signal decay to the model with three parameters (*S*_0_, $R_{2corr}^{*}$, and Δ*B*_0_), referred to as the three-parameter model herein. Nonetheless, with an additional parameter (Δ*B*_0_), the three-parameter model becomes more sensitive to noise at low SNR [17], particularly when the number of echo images is small. An initial estimate of Δ*B*_0_ based on phase images [19,20,21,22] or a separate data acquisition [18] has been used to improve the $R_{2corr}^{*}\;$ estimate, but this requires additional processing of phase images and may increase the scan time.

The purpose of this study is to present an improved postprocessing method to estimate the $R_{2}^{*}$ in the mouse brain at 7T, which mitigates the cross-slice Δ*B*_0_ effect while reducing sensitivity to noise. Based on the three-parameter model and an assumption of the smoothness of the Δ*B*_0_ map, this study presents a two-stage fitting procedure to generate a less noisy estimate of $R_{2}^{*}$. The assumption of the smoothness of the *B*_0_ and Δ*B*_0_ maps has been utilized previously to reduce the noise effect on the Δ*B*_0_ map for the $R_{2}^{*}$ correction in the human brain and liver [21,22]. The novelty of the presented two-stage fitting procedure is that the Δ*B*_0_ map is directly measured by fitting the magnitude images of the mouse brain, followed by the application of a smoothing filter to reduce the noise effects on the measured Δ*B*_0_ map. By eliminating the need to process phase images, our method may help simplify the image processing workflow and provide more flexibility when phase images are unavailable or when obtaining an accurate estimate of *B*_0_ maps through phase images is challenging. We demonstrate the feasibility of the presented method using in vivo mouse experiments and simulations.

## 2. Materials and Methods

### 2.1. In Vivo $R_{2}^{*}$ Measurements

MRI scans were performed following the protocol approved by the Institutional Animal Care and Use Committees (IACUC) at the University of Iowa (IACUC Protocol #2112263). A total of 8 healthy mice (1 female and 7 males; 2–6 months of age) were imaged on a 7 Tesla Discovery MR901 system (GE Healthcare, Milwaukee, WI, USA) using a body transmit coil and a 2-channel mouse brain receiver coil. The animals were sedated with isoflurane during the session. The imaging protocol included a vendor-supplied high-order *B*_0_ shimming routine, followed by a FIESTA sequence for anatomical T_2_-weighted images (in-plane resolution of 104 µm^2^, slice thickness = 160 µm, pixel bandwidth = 326 Hz, flip angle = 30°, TE/TR = 3/6.1 ms, number of averages = 4, and scan time of 9 min and 28 s) and a 2D multi-echo GRE sequence for the $R_{2}^{*}$ measurements (in-plane resolution of 156 µm^2^, slice thickness = 500 µm, 18 axial slices, pixel bandwidth = 244 Hz, flip angle = 60°, TR = 1000 ms, 6 TEs of 2.5–22.5 ms in increments of 4 ms, number of averages = 2, and scan time of 4 min and 24 s).

### 2.2. Data Fitting

Data fitting was performed on the magnitude images in DICOM format that were reconstructed using a vendor-supplied image reconstruction routine. The reconstructed images had an image voxel size of 78 × 78 × 500 µm^3^.

Voxel-wise fitting was performed using the proposed two-stage fitting procedure as described below:

In the first stage of fitting, the signals in each image voxel of six-echo GRE images were fitted with the three-parameter model [17]:

(1) $$S\left(TE\right)=S_{0}\cdot e^{-R_{2corr}^{*}TE}\cdot sinc(\frac{\gamma {∆B}_{0}TE}{2})$$

where TE is the echo time of GRE images, $S_{0}$ is the signal at TE = 0, *γ* is the gyromagnetic ratio, and $∆B_{0}=G_{z}\cdot z_{0}$ ($G_{z}$ is the constant z-direction gradient and $z_{0}$ is the slice thickness). Due to the additional parameter (Δ*B*_0_), the three-parameter model was sensitive to noise and generated noisy estimates of *γ*Δ*B*_0_ and $R_{2corr}^{*}$. By assuming that the *γ*Δ*B*_0_ is slowly varying on the x-y plane, a 2D Gaussian filter with a standard deviation (*σ_gaussian_*) of 390 µm, which was the length of five image pixels and around 2.5 times the in-plane image resolution, was applied to the *γ*Δ*B*_0_ map to reduce the effect of noise and generate a smoothed *γ*Δ*B*_0_ map (*γ*Δ*B*_0*smooth*_).

In the second stage of fitting, given the *γ*Δ*B*_0*smooth*_ map, the multi-echo GRE signals of each image voxel were divided by the sinc weighting function ($sinc(\gamma {∆B}_{0smooth}TE/2$)) in Equation (1) to remove the Δ*B*_0_ effect. Following the division, the signals were fitted by the monoexponential model to obtain the $R_{2corr\_t}^{*}$ with a reduced sensitivity to noise.

For comparison, the monoexponential models were also fitted to the six-echo GRE signals to generate the $R_{2}^{*}$ map without correction ($R_{2nocorr}^{*}$). All of the fittings were performed using the trust-region-reflective algorithm [27] in Matlab R2023b (Mathworks, Inc.). The upper bound of *γ*Δ*B*_0_ was set to 88 Hz given the longest echo time of 22.5 ms [17]. The upper bound of $R_{2}^{*}$ was set to 100 Hz.

Goodness-of-fit was evaluated using the reduced chi-square statistic ($\chi_{\nu }^{2}$) [28]. $\chi_{\nu }^{2}$ quantifies the sum of squares of the residuals normalized by the degrees of freedom of the model and the noise variance of the GRE images. A 95% confidence interval was defined as $\chi_{\nu }^{2}$ < 2.4 for a two-parameter model (the monoexponential model) and as $\chi_{\nu }^{2}$ < 2.6 for a three-parameter model. The Akaike information criterion (AIC) [29] was also used to compare the relative goodness-of-fit between the different fitting methods.

### 2.3. Regions of Interest Analysis

The regions of interest (ROIs), including the corpus callosum, internal capsule, somatosensory cortex, caudo-putamen, thalamus, and lateral ventricle, were selected to evaluate the $R_{2}^{*}$ measurements. The ROIs were extracted from the structural labels of the P56 Mouse Brain atlas images [30,31,32]. These structural labels were brought into the individual GRE image space using a non-linear co-registration between the P56 Mouse Brain atlas images and individual anatomical T_2_-weighted images, followed by the linear co-registration between the individual anatomical T_2_-weighted images and the GRE images. All of the image co-registrations were performed using ANTs [32]. The ROIs on the GRE images were visually examined and adjusted for each mouse and were applied to the $R_{2}^{*}$ maps. The within-ROI mean and standard deviation (SD) values of the $R_{2}^{*}$ measurements were computed for each ROI. The image voxels with a poor fitting ($\chi_{\nu }^{2}$ values outside the 95% confidence interval) were excluded from the ROI analysis.

### 2.4. Effect of the Smoothing Kernel Size

To investigate the effect of the smoothing kernel (*σ_gaussian_*) of the 2D Gaussian filter applied to the *γ*Δ*B*_0_ map, four different values of the *σ_gaussian_*, 234, 390, 546, and 702 µm, were used to smooth the *γ*Δ*B*_0_ map for the two-stage fitting procedure. The effect of the different smoothing kernels on $R_{2corr\_t}^{*}$ was evaluated.

### 2.5. Statistics

Statistical analysis was performed to study (1) whether the fitting error, quantified by the AIC, of the monoexponential model is reduced by the application of the three-parameter model and two-stage fitting procedure; (2) whether the within-ROI SD of $R_{2corr}^{*}$ using the three-parameter model is higher than that of the $R_{2nocorr}^{*}$ of the monoexponential model due to the increased sensitivity to noise; and (3) whether the within-ROI SD of the $R_{2corr}^{*}$ is reduced by the application of the two-stage fitting procedure.

To address the above three questions, the one-tailed Wilcoxon signed-rank test was used for the comparisons, resulting in a total of 24 comparisons within the six ROIs. The significance level was adjusted for multiple comparisons using the false discovery rate [33]; *p*-value < 0.0078.

### 2.6. Simulations

To study the noise effects on the $R_{2}^{*}$ measurements in the presence of a cross-slice Δ*B*_0_ effect, representative in vivo multi-echo GRE signals were simulated using Equation (1) with the parameters $R_{2}^{*}$ of 30 Hz, 6 TEs of 2.5–22.5 ms in increments of 4 ms, and $S_{0}$ of 50. *γ*Δ*B*_0_ was increased from 1 to 45 Hz to reflect the range of measured γΔ*B*_0_ in the selected ROIs of the in vivo mouse brain. Rician noise was added to the signals with an SNR of 50, the average measured SNR of the in vivo mouse brain’s 1st echo image. The procedure was repeated 1000 times to generate 1000 sets of noisy signals. One thousand sets of simulated noisy signals were fitted with the two-stage fitting procedure, monoexponential model, and three-parameter model as described in the Section 2.2. For the two-stage fitting procedure, 1000 measurements of *γ*Δ*B*_0_ were obtained through three-parameter model fitting and were smoothed to generate the *γ*Δ*B*_0*smooth*_ using a 1D Gaussian filter. The *σ_gaussian_* of the 1D Gaussian filter was set to 25 data points to match the square kernel *σ_gaussian_* (5 × 5 image pixels) used for the 2D Gaussian filter. Given the true values of $R_{2}^{*}$ and *γ*Δ*B*_0_, the accuracies of the measured $R_{2}^{*}$ and *γ*Δ*B*_0_ were evaluated using the root mean square error (RMSE). Moreover, the effect of the SNR on the $R_{2}^{*}$ measurements was investigated by changing the SNR of the simulated signals from 20 to 100 and evaluating the accuracies of the measured $R_{2}^{*}$ and *γ*Δ*B*_0_ at different SNR levels.

## 3. Results

**Figure 1:**
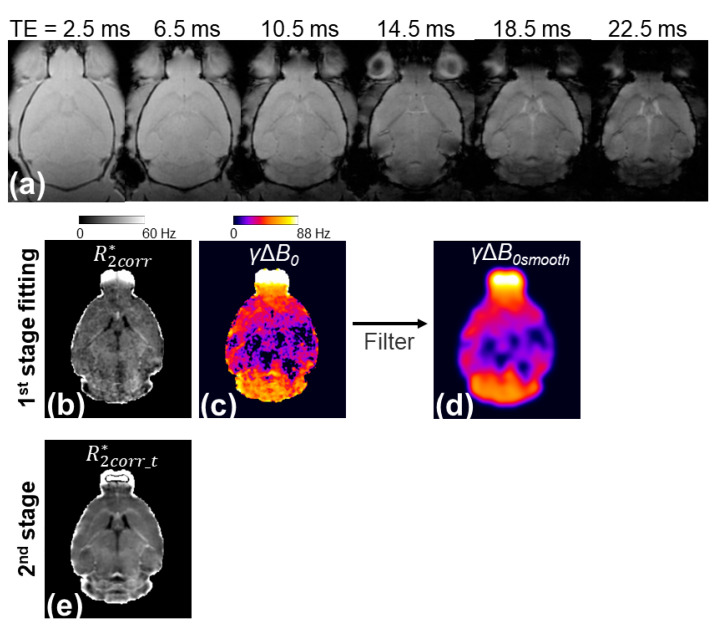
Illustration of the two-stage fitting procedure. In the first stage of fitting, the voxel-wise multi-echo GRE signals shown in (**a**) were fitted with the three-parameter model (Equation (1)) to generate corrected $R_{2}^{*}$ ($R_{2corr}^{*}$) (**b**) and *γ*Δ*B*_0_ maps (**c**). Based on the assumption that the *γ*Δ*B*_0_ map is smooth on the x-y plane, a 2D Gaussian filter with a σ_gaussian_ of 390 µm was applied to the *γ*Δ*B*_0_ map to generate *γ*Δ*B*_0*smooth*_ (**d**). In the second stage of fitting, the multi-echo GRE signals of each image voxel were divided by the $sinc(\gamma {∆B}_{0smooth}TE/2$)) and were then fit with the monoexponential model to generate the $R_{2corr\_t}^{*}$ map with reduced sensitivity to noise (**e**).

Figure 1 illustrates the workflow of the presented two-stage fitting procedure. The *γ*Δ*B*_0_ map generated by the three-parameter fit showed a large Δ*B*_0_ in regions with large magnetic susceptibility changes, such as the olfactory bulb, entorhinal cortex, and cerebellum, but it was noisy (Figure 1c). A smoothing kernel was applied to the *γ*Δ*B*_0_ map to reduce the noise effects while maintaining the spatial variation of Δ*B*_0_ (Figure 1d). The *γ*Δ*B*_0*smooth*_ map was applied to the three-parameter model to remove the Δ*B*_0_ effect from the signal and reduce the unknown parameters from three to two, thereby generating a less noisy $R_{2corr\_t}^{*}$ map (Figure 1e).

**Figure 2:**
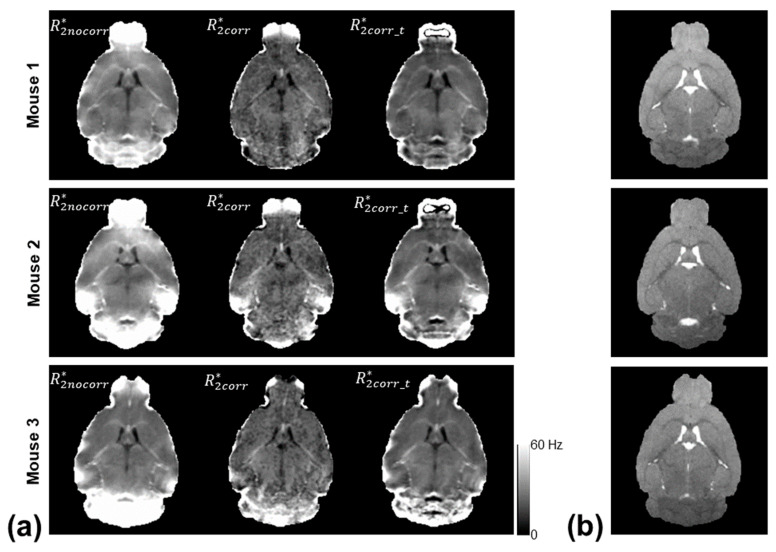
(**a**) Representative $R_{2}^{*}$ measurements of three mice using the monoexponential model ($R_{2nocorr}^{*}$), three-parameter model ($R_{2corr}^{*}$), and two-stage fitting procedure ($R_{2corr\_t}^{*}$), respectively, along with the anatomical T_2_-weighted images (**b**) as a reference.

**Figure 3:**
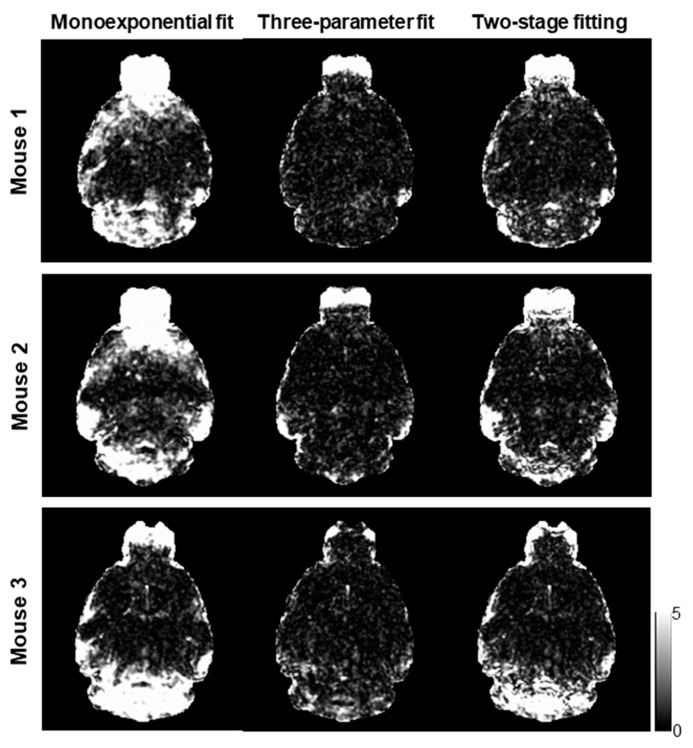
$\chi_{\nu }^{2}$ maps corresponding to the voxel-wise fittings using the monoexponential model, three-parameter model, and two-stage fitting procedure, respectively, to generate the $R_{2}^{*}$ measurements as shown in Figure 2.

Figure 2 shows the computed $R_{2nocorr}^{*}$, $R_{2corr}^{*}$, and $R_{2corr\_t}^{*}$ maps of three mice using the monoexponential model, three-parameter model, and two-stage fitting procedure. Figure 3 shows the corresponding $\chi_{\nu }^{2}$ maps using the three fitting methods. $R_{2nocorr}^{*}$ showed the Δ*B*_0_-induced increases in the regions near air–tissue interfaces, where fitting residuals of the monoexponential fit were elevated. These Δ*B*_0_ effects were consistently mitigated on the $R_{2corr}^{*}$ and $R_{2corr\_t}^{*}$ maps. Furthermore, the noise effect on the $R_{2corr}^{*}$ maps was mitigated on the $R_{2corr\_t}^{*}$ maps without compromising the contrast of the brain structure.

**Figure 4:**
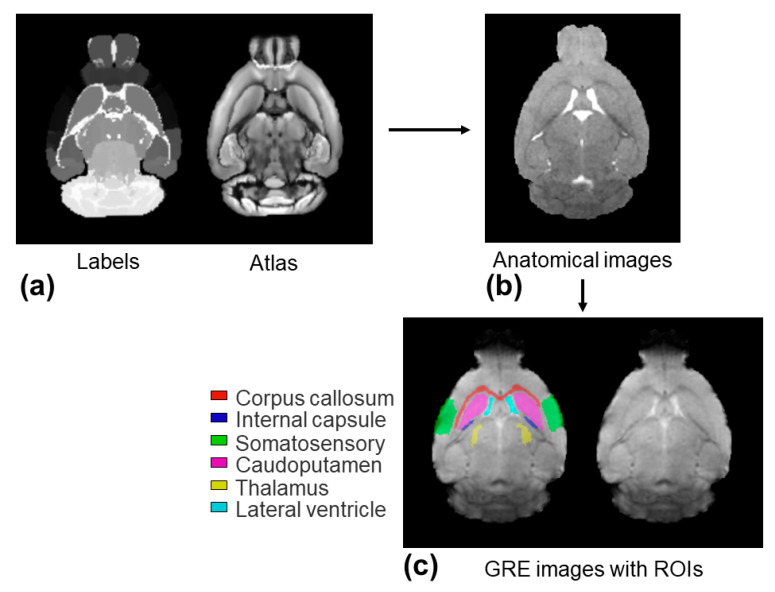
Illustration of the workflow of image co-registrations for the ROI analysis. Firstly, structural labels and the P56 Mouse Brain atlas images shown in (**a**) were brought into the space of individual anatomical images shown in (**b**) through a non-linear co-registration. Secondly, they were brought into the space of individual GRE images through a linear co-registration. (**c**) The selected six ROIs extracted from the structural labels on the individual GRE image space. ROIs were manually adjusted before they were applied to the $R_{2}^{*}$ maps for quantification analysis.

**Figure 5:**
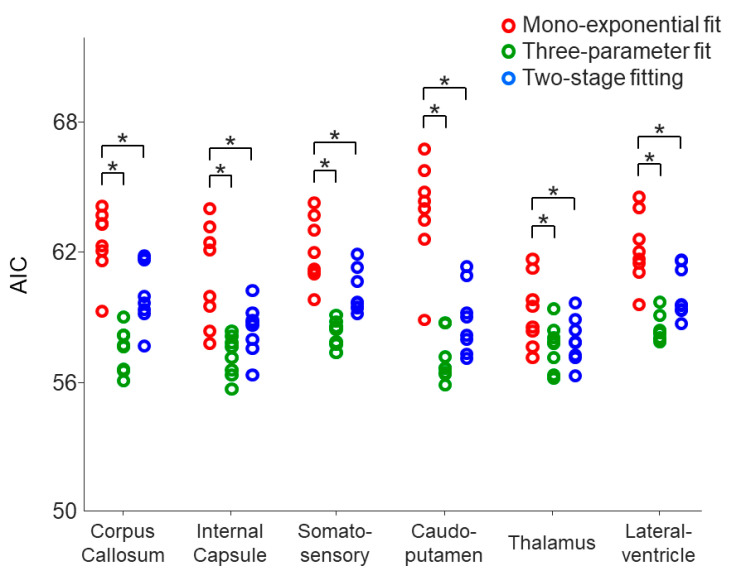
Comparison of the within-ROI mean AIC values on the eight mice using the monoexponential model, three-parameter model, and two-stage fitting procedure. * indicates that the AIC of the monoexponential model was significantly higher than that of the three-parameter model or two-stage fitting procedure. The comparisons were evaluated using the one-tailed Wilcoxon signed rank test (*p* < 0.0078).

Further quantitative analysis on the six selected ROIs was achieved through an image co-registration workflow, as shown in Figure 4. The AIC values of the monoexponential model were significantly reduced by using the three-parameter model and two-stage fitting procedure in all of the selected ROIs (*p* = 0.0039–0.0078) (Figure 5), suggesting that both the three-parameter model and two-stage fitting procedure are preferred to the monoexponential model in describing the data of the six ROIs.

**Figure 6:**
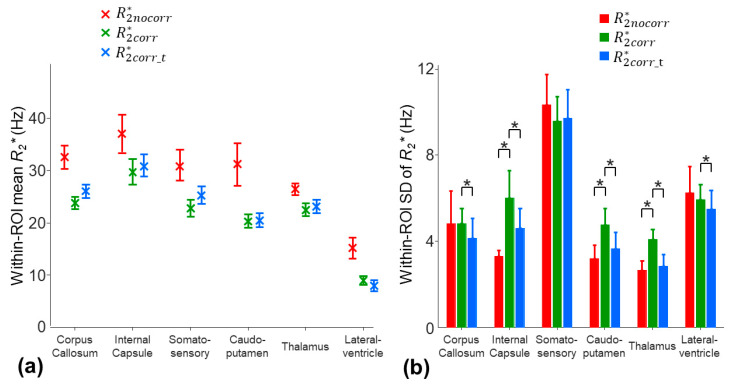
Comparison of the within-ROI mean (**a**) and SD (**b**) values of the $R_{2}^{*}$ measurements on the eight mice using the monoexponential model ($R_{2nocorr}^{*}$), three-parameter model ($R_{2corr}^{*}$), and two-stage fitting procedure ${(R}_{2corr\_t}^{*}$). * in (**b**) indicates that the SD of the $R_{2corr}^{*}$ was significantly higher than that of the $R_{2nocor}^{*}$ or $R_{2corr\_t}^{*}$. The comparisons were evaluated using the one-tailed Wilcoxon signed-rank test (*p <* 0.0078).

The averaged inter-subject SD of the mean $R_{2nocorr}^{*}$ across the ROIs was decreased from 2.7 Hz to 1.4 and 1.5 Hz by using the three-parameter model and two-stage fitting procedure, respectively (Figure 6a). Nonetheless, the within-ROI SD of the $R_{2corr}^{*}$ using the three-parameter model was significantly higher than that of the $R_{2nocorr}^{*}$ of the monoexponential model in the internal capsule, caudo-putamen, and thalamus regions (*p* = 0.0039) (Figure 6b). The higher within-ROI SD of the $R_{2corr}^{*}$ was partially contributed by the increased noise sensitivity due to over-fitting. With the two-stage fitting procedure, the within-ROI SD of the $R_{2corr}^{*}$ was significantly reduced by 7.7–30.2% in all the ROIs, except for the somatosensory cortex region (*p* = 0.0039–0.0078) (Figure 6b).

**Figure 7:**
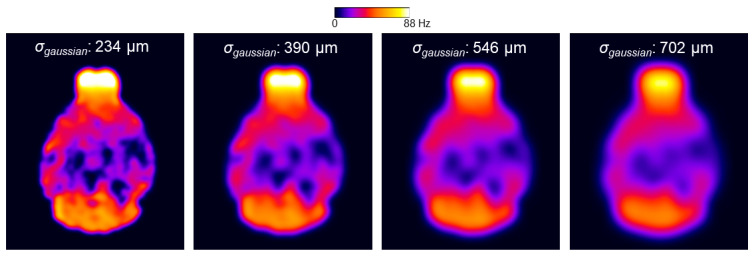
Illustration of the *γ*Δ*B*_0*smooth*_ map for the two-stage fitting procedure using different smoothing kernels (the *σ_gaussian_*: 234, 390, 546, and 702 µm).

For the two-stage fitting procedure, the application of different smoothing kernels to the *γ*Δ*B*_0_ map is illustrated in Figure 7.

**Figure 8:**
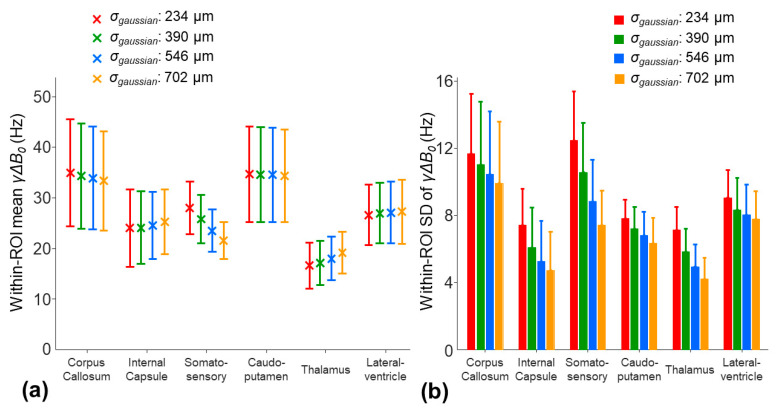
The effect of the different smoothing kernels (the *σ_gaussian_*: 234, 390, 546, and 702 µm) on the within-ROI mean (**a**) and SD (**b**) values of the *γ*Δ*B*_0*smooth*_ map for the eight mice using the two-stage fitting procedure.

**Figure 9:**
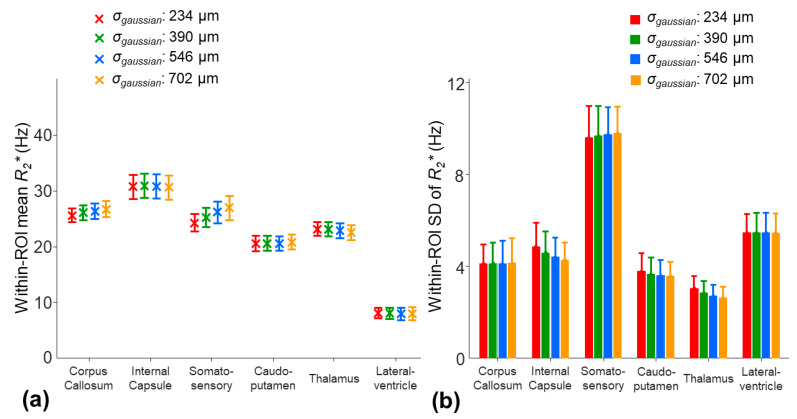
The effect of the different smoothing kernels (the *σ_gaussian_*: 234, 390, 546, and 702 µm) on the within-ROI mean (**a**) and SD (**b**) values of the $R_{2corr\_t}^{*}$ for the eight mice using the two-stage fitting procedure.

The application of a larger smoothing kernel (σ_gaussian_ increased from 234 to 702 µm) led to decreases in the within-ROI SD of the *γ*Δ*B*_0_ in all the ROIs (Figure 8b). Among the selected ROIs, the smoothing procedure had the largest impact on the mean *γ*Δ*B*_0_ in the somatosensory region. The mean *γ*Δ*B*_0_ was decreased by 6.5 Hz in the somatosensory region, whereas the changes in the mean *γ*Δ*B*_0_ were less than 2.7 Hz in other ROIs (Figure 8a). This potential underestimate of the *γ*Δ*B*_0_ in the somatosensory cortex region using a larger smoothing kernel resulted in an increased mean $R_{2corr\_t}^{*}$ by 11.4% (Figure 9a). In other ROIs, the changes in the mean $R_{2corr\_t}^{*}$ were less than 5%. On the other hand, a larger smoothing kernel led to the reduced noise sensitivity of the $R_{2corr\_t}^{*}$. The within-ROI SD of the $R_{2corr\_t}^{*}$ was decreased by 12.3%, 5.6%, and 14% in the internal capsule, caudo-putamen, and thalamus regions, respectively (Figure 9b).

**Figure 10:**
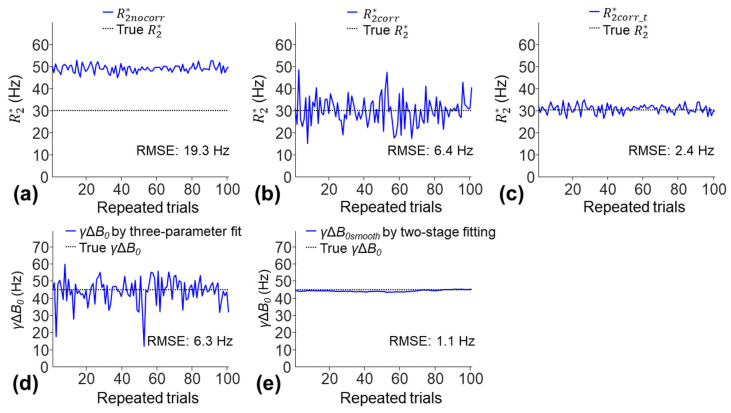
Illustration of the $R_{2}^{*}$ (**a**–**c**) and *γ*Δ*B*_0_ (**d**,**e**) measurements using simulations with a cross-slice Δ*B*_0_ effect. One-hundred sets of noisy signals were generated using the three-parameter model (Equation (1)) with an SNR of 50, $R_{2}^{*}$ of 30 Hz, 6 TEs of 2.5–22.5 ms in increments of 4 ms, and *γ*Δ*B*_0_ of 45 Hz over 100 repeated trials. They were fitted with the monoexponential model, three-parameter model, and two-stage fitting procedure to generate the $R_{2}^{*}$ measurements (**a**–**c**). The *γ*Δ*B*_0_ measurements in (**d**) were obtained through the three-parameter fit. They were smoothed by a 1D Gaussian filter with a *σ_gaussian_* of 25 data points to generate *γ*Δ*B*_0*smooth*_ (**e**) for the two-stage fitting procedure.

The simulations in the presence of a cross-slice Δ*B*_0_ effect (true γΔ*B*_0_ of 45 Hz) were performed to study the accuracies of the $R_{2}^{*}$ and *γ*Δ*B*_0_ measurements. The $R_{2nocorr}^{*}$ had the highest RMSE of 19.3 Hz due to an overestimate of $R_{2}^{*}$ (Figure 10a). The $R_{2corr}^{*}$ showed an improved estimate of $R_{2}^{*}$ (RMSE of 6.4 Hz) but had a high SD due to increased noise sensitivity; the mean ± SD of $R_{2corr}^{*}$ was 30.3 ± 6.5 Hz (Figure 10b). The $R_{2corr\_t}^{*}$ was the most accurate (lowest RMSE of 2.4 Hz); the mean ± SD of $R_{2corr\_t}^{*}$ was 31 ± 2.4 Hz (Figure 10c). The *γ*Δ*B*_0*smooth*_ measured using the two-stage fitting procedure was more accurate than the *γ*Δ*B*_0_ measured using the three-parameter fit (RMSE: 1.1 Hz versus 6.3 Hz).

**Figure 11:**
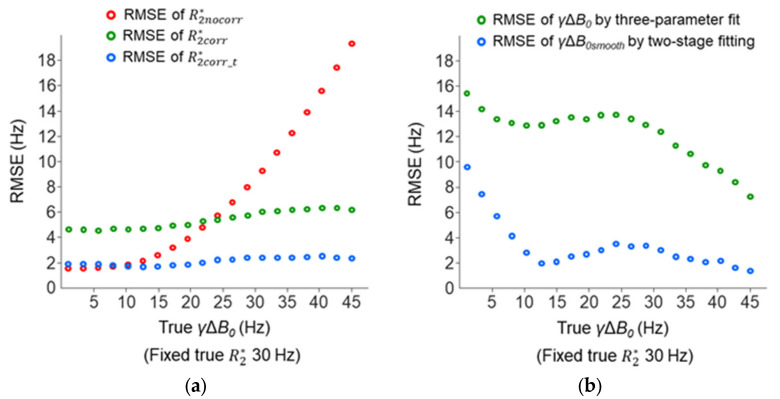
The RMSE of $R_{2}^{*}$ (**a**) and *γ*Δ*B*_0_ (**b**) measurements across the different true *γ*Δ*B*_0_ values used in the simulations as described in Figure 10. Here, one thousand repeated trials were used to generate noisy signals to reduce the variability in the RMSE.

Further simulations were performed to study the accuracies of the $R_{2}^{*}$ and *γ*Δ*B*_0_ measurements with a varied cross-slice Δ*B*_0_ effect (true γΔ*B*_0_ increased from 1 to 45 Hz). The $R_{2nocorr}^{*}$ became inaccurate with an increasing Δ*B*_0_ effect (RMSE change: 1.6–19.3 Hz), whereas the accuracies of $R_{2corr}^{*}$ and $R_{2corr\_t}^{*}$ were more consistent (RMSE changes: 4.6–6.4 Hz and 1.7–2.6 Hz) (Figure 11a). The $R_{2corr\_t}^{*}$ was more accurate than $R_{2corr}^{*}$ and was nearly as accurate as $R_{2nocorr}^{*}$ when the Δ*B*_0_ effect was small (true *γ*Δ*B*_0_ < 10 Hz). The *γ*Δ*B*_0*smooth*_ measured by the two-stage fitting procedure was more accurate than the *γ*Δ*B*_0_ measured by the three-parameter fit (Figure 11b). The accuracy of the *γ*Δ*B*_0*smooth*_ decreased when the Δ*B*_0_ effect was small (true *γ*Δ*B*_0_ < 10 Hz), but this had little impact on the accuracy of $R_{2corr\_t}^{*}$.

**Figure 12:**
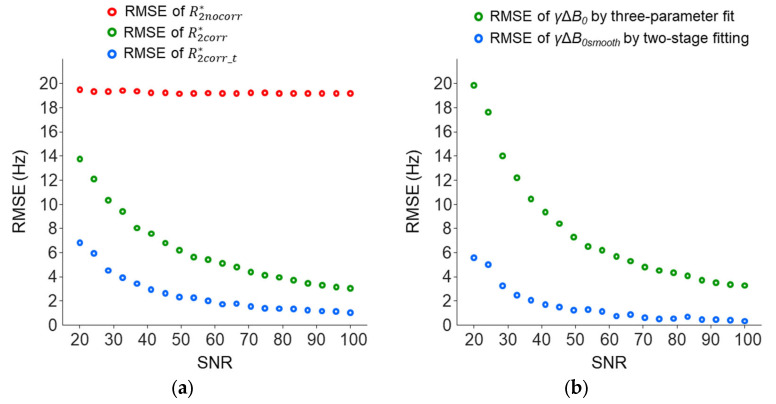
The RMSE of the $R_{2}^{*}$ (**a**) and *γ*Δ*B*_0_ (**b**) measurements across the different SNRs used in the simulations with a true $R_{2}^{*}$ of 30 Hz and true *γ*Δ*B*_0_ of 45 Hz as described in Figure 10. Here, one thousand repeated trials were used to generate noisy signals to reduce the variability in the RMSE.

Simulations were also performed to study the dependence of the accuracies of the $R_{2}^{*}$ and *γ*Δ*B*_0_ measurements on the SNR. With a true *γ*Δ*B*_0_ set to 45 Hz, the RMSE of the $R_{2nocorr}^{*}$ was mainly contributed by the cross-slice Δ*B*_0_ effect and showed a smaller dependence on the SNR levels; RMSE change: 19.3–19.7 Hz versus 3.2–14.1 Hz for $R_{2corr}^{*}$ and 1.1–6.9 for $R_{2corr\_t}^{*}$ (Figure 12a). Across the SNR levels, $R_{2corr\_t}^{*}$ was more accurate than $R_{2nocorr}^{*}$ and $R_{2corr}^{*}$. The *γ*Δ*B*_0*smooth*_ measured using the two-stage fitting procedure was more accurate than the *γ*Δ*B*_0_ measured using the three-parameter fit (Figure 12b).

## 4. Discussion

The Δ*B*_0_ effects include a deviation of the GRE signal decay from the monoexponential model, a potential overestimate of the $R_{2}^{*}$, and increased inter-subject SD of $R_{2}^{*}$. With imaging data from eight mouse brains, we have shown that these Δ*B*_0_ effects were effectively mitigated using the three-parameter model in the selected ROIs. We have further demonstrated that the noise-related within-ROI SD of $R_{2corr}^{*}$ was significantly reduced by up to 30.2% using the two-stage fitting procedure. Moreover, simulations of $R_{2}^{*}$ measurements in the presence of a cross-slice Δ*B*_0_ effect and noise have demonstrated that $R_{2corr\_t}^{*}$ was more accurate than $\;R_{2corr}^{*}$. Taken together, these results support the use of the two-stage fitting procedure to mitigate Δ*B*_0_ effects and reduce noise sensitivity for $R_{2}^{*}$ measurement in the mouse brain.

Applying a smoothing filter to the *γ*Δ*B*_0_ map for the two-stage fitting procedure assumes that the Δ*B*_0_ is slowly varying on the x-y plane. Based on our results, this assumption may be valid in the selected corpus callosum, internal capsule, caudo-putamen, thalamus, and lateral ventricle regions of the mouse brain, where the application of the two-stage fitting procedure yields a significant reduction in the within-ROI SD of $R_{2corr}^{*}$ by 7.7 to 30.2%. For the selected ROIs in the deep brain structure, including the internal capsule, caudo-putamen, and thalamus regions, the within-ROI SD of the $R_{2corr\_t}^{*}$ in these ROIs was further reduced with a larger smoothing kernel applied to the *γ*Δ*B*_0_ map. However, the assumption of a slow in-plane variation in *γ*Δ*B*_0_ is invalid in regions with a rapidly varying *γ*Δ*B*_0_ on the x-y plane, such as the somatosensory cortex region close to air–tissue interfaces and with large magnetic susceptibility changes. In these regions, the *γ*Δ*B*_0_ map as well as $R_{2corr\_t}^{*}$ showed a strong dependence on the smoothing kernel. Furthermore, the application of a larger smoothing kernel only led to small changes (<2%) in the within-ROI SD of $R_{2corr\_t}^{*}$, indicating no benefits of using a larger smoothing kernel in these regions. In the presence of a fast in-plane variation in *γ*Δ*B*_0_, applying a smoothing filter may lead to an underestimate of *γ*Δ*B*_0_ and an overestimate of $R_{2corr\_t}^{*}$ [17]. This $R_{2corr\_t}^{*}$ change may in turn increase the within-ROI SD of $R_{2corr\_t}^{*}$ and offset the benefit of the two-stage fitting procedure to reduce noise sensitivity. Considering the tradeoff, this study used a smoothing kernel of 390 µm, around 2.5 times the in-plane image resolution, for the two-stage fitting procedure. Importantly, we observed only a moderate dependence of the mean $R_{2corr\_t}^{*}$ on the smoothing kernel in the selected ROIs; the changes were less than 11.4%.

In our study, the *γ*Δ*B*_0_ map was measured through the fitting of the six echo magnitude images with the three-parameter model that assumes a linear Δ*B*_0_ across a slice. The assumption of a linear Δ*B*_0_ implies a slowly varying Δ*B*_0_ across a slice and may be invalid in regions with a rapidly varying cross-slice Δ*B*_0_, such as a high Δ*B*_0_. In our study, regions with a measured *γ*Δ*B*_0_ larger than 60 Hz, such as the olfactory bulb, entorhinal cortex, and cerebellum, showed a potential overestimate of the $R_{2corr}^{*}$ and thus an inaccurate estimate of the *γ*Δ*B*_0_ map using the three-parameter model (Figure 2). An alternative approach to measuring the *γ*Δ*B*_0_ map is using phase images [19,20,21,22]. The phase image-based approach typically requires phase unwrapping procedures and an assumption of a smoothed *B*_0_ on the x-y plane to reliably measure the *B*_0_ map. Thus, the phase-based approach remains limited in the presence of a rapidly varying Δ*B*_0_ across a slice or at high fields with a fast phase evolution. Our study demonstrates the feasibility of measuring the *γ*Δ*B*_0_ map through the fitting to magnitude images, omitting the need for processing the phase images.

The use of magnitude images to measure the *γ*Δ*B*_0_ map relies on the sinc function-weighted signal decay, which is particularly pronounced at long echo times. When the Δ*B*_0_ effect is small, e.g., *γ*Δ*B*_0_ < 10 Hz, the signal attenuation may be too small to reliably measure the *γ*Δ*B*_0_. As shown in our simulation, the accuracy of the *γ*Δ*B*_0*smooth*_ decreased when the true *γ*Δ*B*_0_ was less than 10 Hz. However, the inaccurate *γ*Δ*B*_0*smooth*_ had little impact on the accuracy of the $R_{2corr\_t}^{*}$ in our simulation. When the Δ*B*_0_ effect is small, the Δ*B*_0_ effect on the signal attenuation may be indifferentiable from the noise, and the accuracy of the *γ*Δ*B*_0*smooth*_ becomes less relevant to the $R_{2corr\_t}^{*}$. On the other hand, phase-based methods may be more sensitive to detecting Δ*B*_0_-induced phase changes even when the Δ*B*_0_ effect is small. This may lead to more accurate estimates of *γ*Δ*B*_0_ and $R_{2}^{*}$.

In addition to the increased within-ROI SD of the $R_{2}^{*}$ measurement, a low SNR could induce bias in $R_{2}^{*}$ measurement. In regions with a rapidly varying cross-slice Δ*B*_0_, the sinc function-weighted signal decay is faster than the monoexponential decay at longer echo times, resulting in low SNR. In our study, the average SNR of the mouse brain’s 1st echo image (TE = 2.5 ms) was around 50. Given a $R_{2}^{*}$ of 30 Hz and the measured *γ*Δ*B*_0_ of 10–50 Hz in our selected ROIs, the SNR of the longest echo signal (TE = 22.5 ms) is around 15–27. However, the SNR at TE = 22.5 ms drops to 3–11 when the *γ*Δ*B*_0_ is increased to 60–80 Hz in the olfactory bulb, entorhinal cortex, and cerebellum regions. This *γ*Δ*B*_0_-induced signal drop at longer echo times may affect the ability of the three-parameter model to characterize the signal decay, likely resulting in an underestimate of *γ*Δ*B*_0_ and an overestimate of $R_{2corr}^{*}$ [17].

The presented two-stage fitting procedure is based on the three-parameter model; therefore, it remains subject to the limitations of the three-parameter model in the presence of a rapidly varying cross-slice Δ*B*_0_ as described above. Considering these limitations, this study focused on the ROIs with a *γ*Δ*B*_0_ less than 50 Hz. To account for a rapidly varying cross-slice Δ*B*_0_, previous studies have used separate data acquisition for a 3D high-resolution *γ*Δ*B*_0_ map [16,18] and a quadratic function to approximate the cross-slice Δ*B*_0_ [16]. However, such approaches increase the scan time and require more fitting parameters to mitigate the Δ*B*_0_ effect.

Our method shares some similarities with previous works by Dong et al. [34] and Tan et al. [35] in seeking the optimized solution for multiple parametric measurements that ensures data consistency and the smoothness of the phase maps. The major difference is that the previous works include the regularization of the loss function to ensure the smoothness of the phase maps, allowing one to address the optimization with more unknowns and more constraints. In contrast, our method ensures data consistency through the voxel-wise fitting, followed by applying a smoothing filter to smooth the phase maps. Solving regularized optimization normally requires multiple iterations and a longer computation time. Our method requires a short computation time but is limited to addressing a simple problem. Another difference is that the work by Tan et al. [35] involves joint image reconstruction and parametric measurements for optimization. This allows one to reconstruct the undersampled k-space data with potential benefits in shortening the scan time and reducing motion artifacts. By contrast, our method only performs parametric measurements on the magnitude images, which are reconstructed using a vendor-supplied image reconstruction routine. Therefore, our method does not require k-space data and can be generally applied to multi-echo GRE images retrospectively.

In this study, multi-echo GRE acquisition was performed using the axial orientation with 18 slices to cover the entire brain. Another common option for 2D acquisition is to use the coronal orientation. However, the axial orientation allows one to cover a larger area of the mouse brain in a 2D slice than the coronal orientation. This is beneficial to our proposed method due to a smaller proportion of an axial image being influenced by large magnetic susceptibility changes near the aural cavity or air–tissue interfaces. Therefore, the assumption of a slow in-plane variation in *γ*Δ*B*_0_ can be applicable to a larger region of the brain, allowing the smoothing filter to effectively reduce the noise effect on the *γ*Δ*B*_0_ map.

This study applied $\chi_{\nu }^{2}$ and AIC to evaluate the goodness-of-fit. For each image voxel, the $\chi_{\nu }^{2}$ was used to determine whether the individual model fits the data considering the noise and the degrees of freedom of the model. Following the exclusion of the image voxels with a poor fit, AIC was applied subsequently to compare the goodness-of-fit of the models within each ROI considering the complexity of the model. Either assessment can eliminate the possibility of over-fitting. Therefore, this study focused on whether the application of the three-parameter model or two-stage fitting procedure improves the monoexponential fit rather than finding the best model to fit the data.

## 5. Conclusions

The presented two-stage fitting procedure reduced the noise-related within-ROI SD of $R_{2corr}^{*}$ in regions with a slow in-plane variation of *γ*Δ*B*_0_. This suggests that it can be used for the three-parameter model to mitigate the cross-slice Δ*B*_0_ effects and reduce noise sensitivity for $R_{2}^{*}$ measurement in the mouse brain. It utilizes fittings of the magnitude images without processing the phase images, thereby helping simplify the image processing workflow.

## Data Availability

Study data are available from the corresponding author upon request.
